# Combined resistance and aerobic training improves lung function and mechanics and fibrotic biomarkers in overweight and obese women

**DOI:** 10.3389/fphys.2022.946402

**Published:** 2022-09-07

**Authors:** Anamei Silva-Reis, Maysa Alves Rodrigues Brandao-Rangel, Renilson Moraes-Ferreira, Thiago Gibson Gonçalves-Alves, Victor Hugo Souza-Palmeira, Helida Cristina Aquino-Santos, Andre Luis Lacerda Bachi, Luis Vicente Franco de Oliveira, Rodrigo Álvaro Brandão Lopes-Martins, Iranse Oliveira-Silva, Regiane Albertini, Claudio Ricardo Frison, Rodolfo P Vieira

**Affiliations:** ^1^ Post-graduation Program in Sciences of Human Movement and Rehabilitation, Federal University of Sao Paulo, São Paulo, Brazil; ^2^ Post-graduation Program in Health Science, University of Santo Amaro, São Paulo, Brazil; ^3^ Post-graduation Program in Human Movement and Rehabilitation, Centro Universitário UniEvangélica, Anápolis, Brazil

**Keywords:** exercise immunology, lung, overweight, obesity, physical training, pulmonary inflammation and fibrosis

## Abstract

**Background:** Obesity impairs lung function and mechanics and leads to low-grade inflammation, but the effects of combined physical exercise (CPE) on that are unknown.

**Methods:** We investigated the effects of 12 weeks of combined physical exercise (aerobic + resistance training), in non-obese (*n* = 12), overweight (*n* = 17), and obese grade I (*n* = 11) women. Lung function and lung mechanics were evaluated. The systemic immune response was evaluated by whole blood analysis and biomarker measurements, while pulmonary fibrotic biomarkers were evaluated in the breath condensate.

**Result:** CPE improved forced vital capacity (FVC) % (*p* < 0.001) and peak expiratory flow (PEF) % (*p* < 0.0003) in the obese group; resistance of the respiratory system (R5Hz) in non-obese (*p* < 0.0099), overweight (*p* < 0.0005), and obese (*p* < 0.0001) groups; resistance of proximal airways (R20Hz) in non-obese (*p* < 0.01), overweight (*p* < 0.0009), and obese (*p* < 0.0001) groups; resistance of distal airways (R5Hz–R20Hz) in non-obese (*p* < 0.01), overweight (*p* < 0.0012), and obese (*p* < 0.0001) groups; reactance of the respiratory system (X5Hz) in non-obese (*p* < 0.01), overweight (*p* < 0.0006), and obese (*p* < 0.0005) groups; impedance of the respiratory system (Z5Hz) in non-obese (*p* < 0.0099), overweight (*p* < 0.0005), and obese (*p* < 0.0001) groups; central resistance (RCentral) in non-obese (*p* < 0.01), overweight (*p* < 0.001), and obese (*p* < 0.0003) groups; and the peripheral resistance (RPeripheral) in non-obese (*p* < 0.03), overweight (*p* < 0.001), and obese (*p* < 0.0002) groups. CPE reduced the pro-fibrotic IGF-1 levels in BC in overweight (*p* < 0.0094) and obese groups (*p* < 0.0001) and increased anti-fibrotic Klotho levels in BC in obese (*p* < 0.0001) groups, and reduced levels of exhaled nitric oxide in overweight (*p* < 0.03) and obese (*p* < 0.0001) groups.

**Conclusion:** CPE improves lung function, mechanics, and pulmonary immune response in overweight and obese grade I women by increasing anti-fibrotic protein Klotho and reducing pro-fibrotic IGF-1.

## Introduction

Obesity has become a pandemic and it is a growing public health problem, especially in developed and developing countries (Kelly et al., 2008). The data provided by the Center for Health Statistics demonstrated that 39.8% of North American adults are obese, with a higher prevalence in women (41.5%) than in men (38%) (Hales et al., 2017). In Brazil, obesity affects 19.8% of Brazilian adults, with 20.7% prevalence in women and 18.7% in men. In addition, 55.7% of the Brazilian population is overweight (Brasil, 2019). Being overweight is the first step to obesity; the development of obesity is associated with several comorbidities which compromise life expectancy, such as cardiovascular diseases, hypertension, and diabetes, and other less investigated health issues, such as respiratory and immunological comorbidities. For instance, reduced levels of adiponectin have been found in obese individuals and are related to decreased insulin sensitivity and increased low-grade inflammation (Moran et al., 2011). In addition, a pre-clinical study demonstrated that obese mice present structural alterations in the lungs, notably in the airways, characterized by airway fibrosis, related to increased levels of insulin-like growth factor 1 (IGF-1) in the lungs (Aquino-Santos et al., 2020). Of note, such fibrotic alterations can be functionally detected in humans by impulse oscillometry, a method used for evaluation of lung mechanics, which has been used in the present study. Interestingly, the anti-inflammatory cytokine IL-10 has been found reduced in obese individuals and it is related to the development of metabolic syndrome in such individuals (Esposito et al., 2003).

The alterations in the respiratory system induced by increases in the body mass, mainly by fat accumulation in the abdominal component of the chest wall, alter the balance among the respiratory pressures, leading to significant changes in ventilation (Zerah et al., 1993). So, the lung function test in obese individuals reveals a typical restrictive lung response, with a reduction in the pulmonary volumes, until 20–30% for the total lung capacity (TLC) and vital capacity (VC), in cases of more severe obesity (Zerah et al., 1993). However, the exact mechanisms involved in the pulmonary alterations induced by obesity are not completely understood.

On the other side, some studies showed that increased concentrations of proinflammatory adipokines, such as leptin and resistin and reduced concentrations of adiponectin, an anti-inflammatory adipokine, are involved in several aspects of comorbidities induced by obesity (Anderson et al., 2016). In fact, such adipokine imbalance also leads to immune dysregulation (Anderson et al., 2016). Furthermore, genetic (Johnson et al., 2013; Speakman, 2013) and energy imbalance caused by a sedentary lifestyle (Mobley et al., 2006) are strongly associated with obesity pathogenesis and obesity comorbidities.

However, the regular practice of physical activity is an important component for controlling and reducing body mass, notably, fat mass (Mobley et al., 2006). Beyond that, physical activity can positively modulate the immune system (Ciolac et al., 2020) and improve lung function (Chlif et al., 2017). However, whether a combined physical training program constituted by aerobic plus resistance training can attenuate the pulmonary and immunological effects of obesity in overweight and obese women are unknown and has been addressed by the present study.

## Methods

All proceedings performed in this study have been approved by the ethical committee of the Federal University of Sao Paulo (registration number 11159619.4.0000.5505) and were performed in accordance with the Declaration of Helsinki.

### Volunteer recruitment and selection

The recruitment was based on sample size calculation, based on a previous study by Perez et al. (2017), which focused on the levels of TNF-α of obese patients before and after bariatric surgery. Considering an error of 0.05 and a significance level of 95%, the total sample size to detect an effect of 0.88 was 24. A total of 41 volunteers (11 women with obesity grade I; 47,36 ± 10,64 years old, BMI 31,98 ± 1,45 kg/m2, 17 overweight women, 47,35 ± 11,75 years old, BMI 27,93 ± 1,67 kg/m2, and 13 non-obese women, 43,5 ± 11,3 years old, BMI 22 ± 1,9 kg/m2) were recruited through a social program in a public sports park in the city of São José dos Campos—SP, Brazil, and were enrolled in the study after agreement and signature of the free consent inform term. So, three groups were studied: non-obese women, overweight women, and obesity grade I women. The inclusion criteria encompassed women of age 30–59 years, into the three BMI classifications, such as non-obese, overweight and grade I obese. The exclusion criteria encompassed musculoskeletal diseases, cardiorespiratory diseases, active smoking, or even ex-smokers (for no longer than 3 years) and performing any type of exercise 1 year before the beginning of the study, characterizing the volunteers as sedentary. In addition, the ones who have not completed at least 80% of all training sessions and do not participate in all evaluations also were excluded from the study. However, we clarify that we have no dropouts during the entire study.

### Aerobic training

Aerobic training was performed on a treadmill, 3x/week, at moderate intensity (70–80% of the maximal heart rate), for 30 min, for 12 weeks (Matsubara et al., 2014).

### Resistance training

Resistance training was constituted of five exercises (deadlift, incline bench press, seated row, abdominal, and dumbbell shoulder press), which took approximately 30 min to be completed. The first week of training was used for familiarization with the exercises. Three sessions per week were performed for 12 weeks. Each exercise was performed using 8–12 repetitions, three sets, and with an interval period of 1–2 min among the sets. The training load was incremented weekly by 2–10% of the initial load every time the volunteers were capable of performing the exercise in the whole extension (in maximal amplitude) for two consecutive sessions. In addition, the following signs of fatigue were observed: tending to fail during the concentric phase of the movement and reduction of the velocity, apnea, and isometric period of contraction (Garber et al., 2011). The following evaluations were performed in the given order before and after the 12 weeks of training, being the evaluation post the 12 weeks performed 24 h after the last exercise session, always from 08:00 a.m until 12:00 a.m. The incremental shuttle walking test (ISWT) was also performed on the morning of the following day (not on the same day as the other evaluations).

### Body composition analysis

After the interview with the volunteers, the first measurement taken was the body composition, analyzed by multi-frequency octopolar bioimpedance (Bioscan 920-II-S, Matron, United Kingdom), always between 08:00–10:00 a.m. (Aquino-Santos et al., 2020).

### Fractional exhaled nitric oxide

The second analysis conducted was the levels of fractional exhaled nitric oxide (FeNO), which were measured using the portable nitric oxide device NOBreath (Bedfont Scientific, United Kingdom) to evaluate pulmonary inflammation (Aquino-Santos et al., 2020).

### Respiratory muscle strength

The third analysis conducted was the measurement of respiratory muscle strength, which was measured by using an analogical manovacuometer (0–300 cmH2O; Murenas, MG, Brazil). Maximal inspiratory pressure (MIP) and expiratory (MEP) pressures were obtained as previously published (Do Nascimento et al., 2015).

### Lung function and mechanics

The fourth analysis conducted was that of lung function, which was evaluated by using a spirometry pre- and post-400 mcg of bronchodilator (salbutamol sulfate) by using the forced maneuver and the Masterscreen spirometer (Jaeger, Germany), using the reference values for the Brazilian population (Duarte et al., 2007). The forced vital capacity (FVC), forced expiratory volume in the first second (FEV1), relation FEV1/FVC, peak expiratory flow (PEF), and maximal expiratory pressure flow at 25, 50, and 75% (MEF 25%, MEF 50%, and MEF 75%) were evaluated (Albuquerque et al., 2015; Aquino-Santos et al., 2020).

Respiratory mechanics was evaluated using the impulse oscillometry system (ios). This method is based on the measurement of resistance of the respiratory systems at different frequencies (i.e., R5 Hz, R20 Hz, R5 Hz–R20 Hz, etc.) which specifically reveals the resistance of total (R5 Hz), proximal (R20 Hz), and distal (R5–R20 Hz) airways. In addition, this method also measures the impedance of the respiratory system (Z5 Hz), the reactance of the respiratory system (X5 Hz), and the resonant frequency (Fres), additionally demonstrating the resistance and elastance of proximal and peripheral lung tissue (Albuquerque et al., 2015; Brashier and Salvi, 2015).

### Pulmonary fibrotic biomarkers

The fifth analysis conducted was the levels of pulmonary fibrotic biomarkers, which were evaluated by measuring the following biomarkers in breath condensate. The breath condensate was collected by using an RT tube (Respiratory Research, TX, United States) according to the manufacturer’s instructions for 15 min with the volunteers breathing at a tidal volume.

The breath condensate samples were stored at -86°C until the measurements of adiponectin, insulin-like growth factor 1 (IGF-1), interleukin 10 (IL-10), and klotho were taken. The measurements were taken using DuoSet ELISA kits (R&D Systems; MN, EUA) and the reads were carried out using the microplate reader Spectramax I3 (Molecular Devices, CA, EUA) (Aquino-Santos et al., 2020).

### Systemic inflammation and immune response

The sixth analysis was of the systemic inflammation and immune response. A total of 5 milliliters of venous blood was collected, using a sterile vacuum tube containing EDTA K3 as the anticoagulant. A total of 25 μL was immediately used for whole blood analysis (platelets and white and red cells) using the automatized Sysmex 800i (Sysmex, Europe GmbH, Germany). The remaining blood was centrifuged at 1,000 g, for 7 min, at 4°C, and the plasma was stored at −86°C until the measurement of adiponectin (DY1065), IL-10 (DY217), IGF-1 (DY291), and Klotho (DY5334-05) were taken using DuoSet ELISA kits (R&D Systems), and the reads were carried out using the microplate reader Spectramax I3 (Molecular Devices, CA, EUA) (Aquino-Santos et al., 2020).

### The functional capacity by incremental shuttle walking test

The seventh analysis was the incremental shuttle walking test (ISWT), which was performed in a 10 m long corridor marked by two cones placed 0.5 m from the end of the course, using a sound device to indicate the progress of the test and the speed changes. The reference equation proposed by Jürgensen et al. was used to calculate the walked distance (WD) (Do Nascimento et al., 2015). This analysis was carried out on the following day as the other analyses described before.

### Statistical analysis

Software GraphPad Prism 5.0 was used to perform the statistical analysis and build the graphs. The distribution of the data was performed using Pearson’s’ test. All data presented parametric distribution, and therefore, were evaluated using one way ANOVA followed by the Newman–Keuls test for multiple comparisons among the groups. A *p* < 0.05 was considered statistically significant. All data were presented as mean ± standard deviation since all data presented normal distribution.

## Results

### Effects of combined training on body composition

**FIGURE 1:**
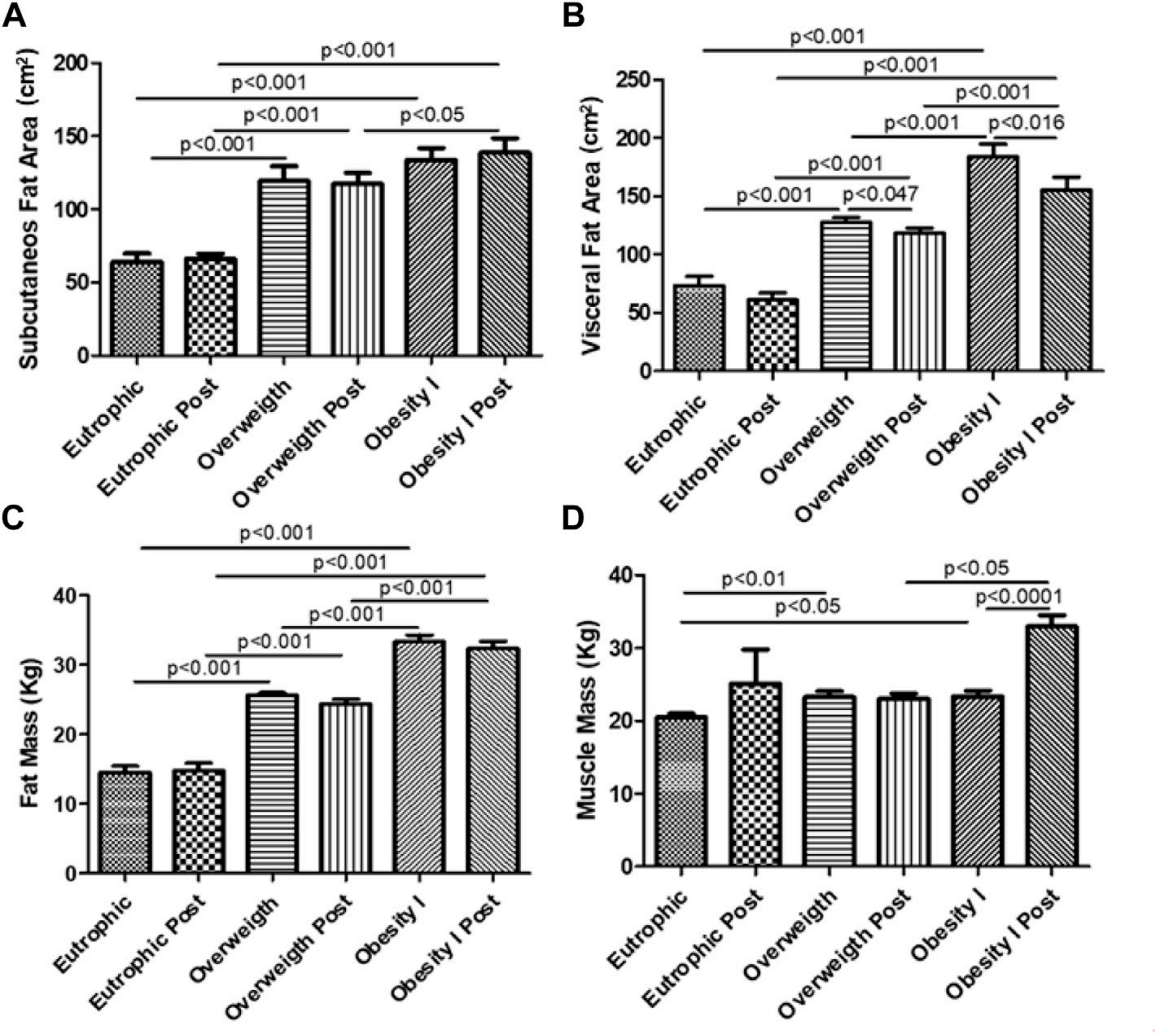
Effects of combined training on body composition. Figure 1A shows the subcutaneous fat area. Figure 1B shows the visceral fat area. Figure 1C shows the total fat mass. Figure 1D shows muscle mass. Paired *t*-test was performed to compare the pre- and post-effects of rehabilitation (i.e., eutrophic non-obese pre x eutrophic non-obese post; overweight pre x overweight post; obesity I pre x obesity I post). A *p* < 0.05 was considered statistically significant. These data presented parametric distribution and were additionally evaluated using one way ANOVA followed by the Newman–Keuls test for multiple comparisons among the groups.

Figure 1 shows the body composition of women submitted to combined training. Figure 1A shows that overweight (*p* < 0.001) and obesity I (*p* < 0.001) groups showed increased subcutaneous fat area compared with the non-obese group and that combined training did not change this parameter. Considering the visceral fat area, Figure 1B shows that the overweight (*p* < 0.001) and obesity I (*p* < 0.001) groups presented an increased amount of visceral fat area compared to the non-obese group. Of note, combined training reduced the visceral fat area in the obesity I (*p* < 0.016) group. However, as shown in Figure 1C, although overweight (*p* < 0.001) and obesity I (*p* < 0.001) groups presented a higher amount of total fat mass than the non-obese group, combined training did not reduce it. In addition, Figure 1D shows that overweight (*p* < 0.01) and obesity I (*p* < 0.05) group presented higher levels of muscle mass than the non-obese group. Furthermore, as shown in Figure 1D, combined training increased muscle mass in the obesity I group (*p* < 0.0001).

### Effects of combined training on the levels of FeNO

**FIGURE 2:**
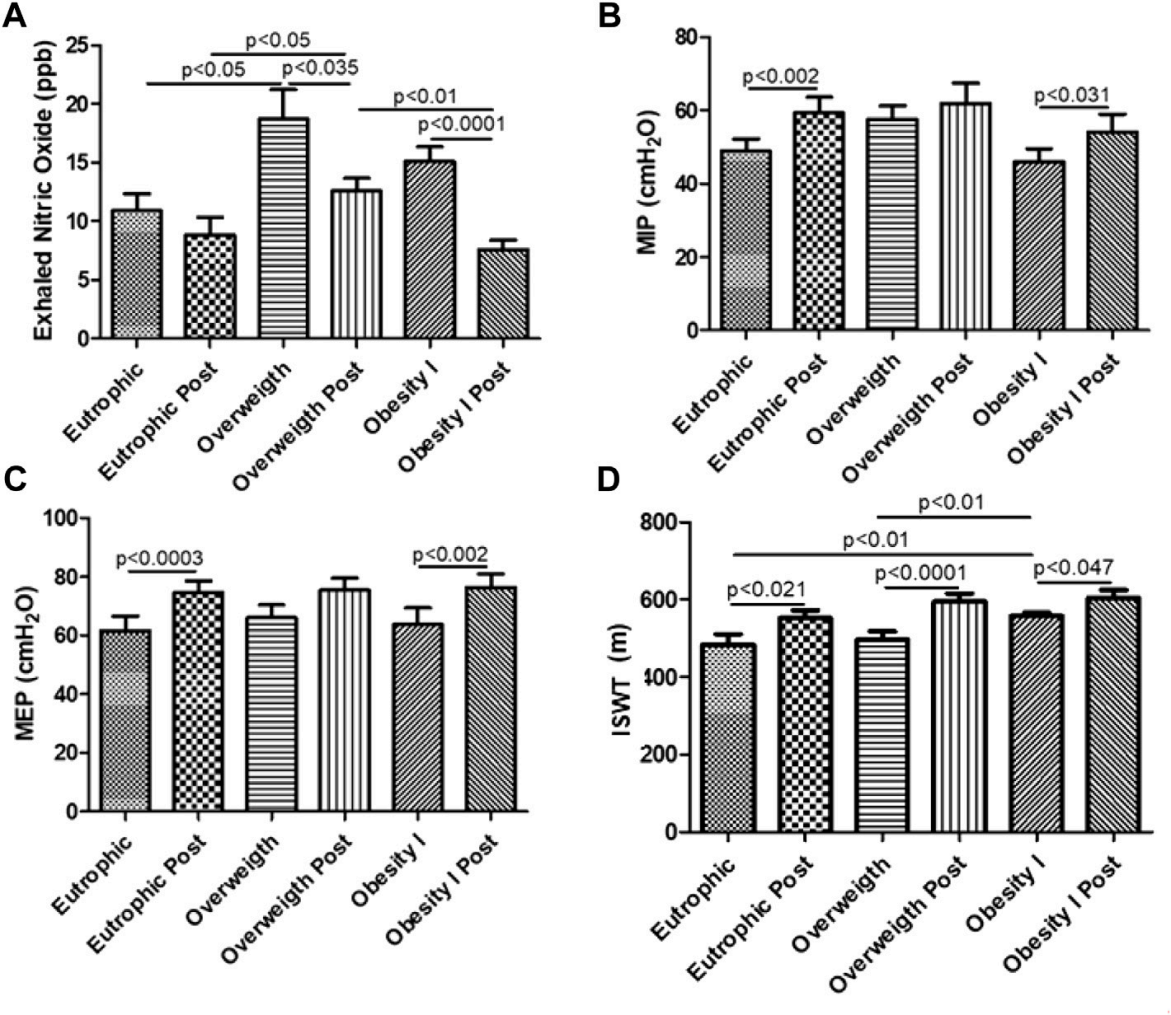
Effects of combined training on the levels of FeNO, MIP, MEP, and ISWT. Figure 2A shows the levels of fractional exhaled nitric oxide (FeNO). Figure 2B shows the maximum inspiratory pressure (MIP). Figure 2C shows the maximum expiratory pressure (MEP). Figure 2D shows the distance reached in ISWT. Paired *t*-test was performed to compare the pre- and post-effects of rehabilitation (i.e., eutrophic non-obese pre x eutrophic non-obese post; overweight pre x overweight post; obesity I pre x obesity I post). A *p* < 0.05 was considered statistically significant. These data presented parametric distribution and were additionally evaluated using one way ANOVA followed by the Newman–Keuls test for multiple comparisons among the groups.

Figure 2A shows that the overweight group (*p* < 0.05) presented higher levels of FeNO than the non-obese group. In addition, Figure 2A also shows that combined training reduced the levels of FeNO in overweight (*p* < 0.05) and obesity I (*p* < 0.0001) groups.

### Effects of combined training on the maximum expiratory and inspiratory pressure

Figure 2B shows that combined training increased the maximum inspiratory pressure (MIP) in the non-obese (*p* < 0.01) and obesity I groups (*p* < 0.05) comparing the pre- versus post-training period. Similar to the MIP, combined training increased the maximum expiratory pressure (MEP) in the non-obese (*p* < 0.01) and obesity I groups (*p* < 0.01).

### Effects of combined training on the functional capacity by the incremental shuttle walking test

Figure 2D shows that combined training increased the distance reached in the incremental shuttle walking test (ISWT) for non-obese (*p* < 0.05), overweight (*p* < 0.05), and obesity I (*p* < 0.05) groups after combined training.

### Effects of combined training on lung function

**FIGURE 3:**
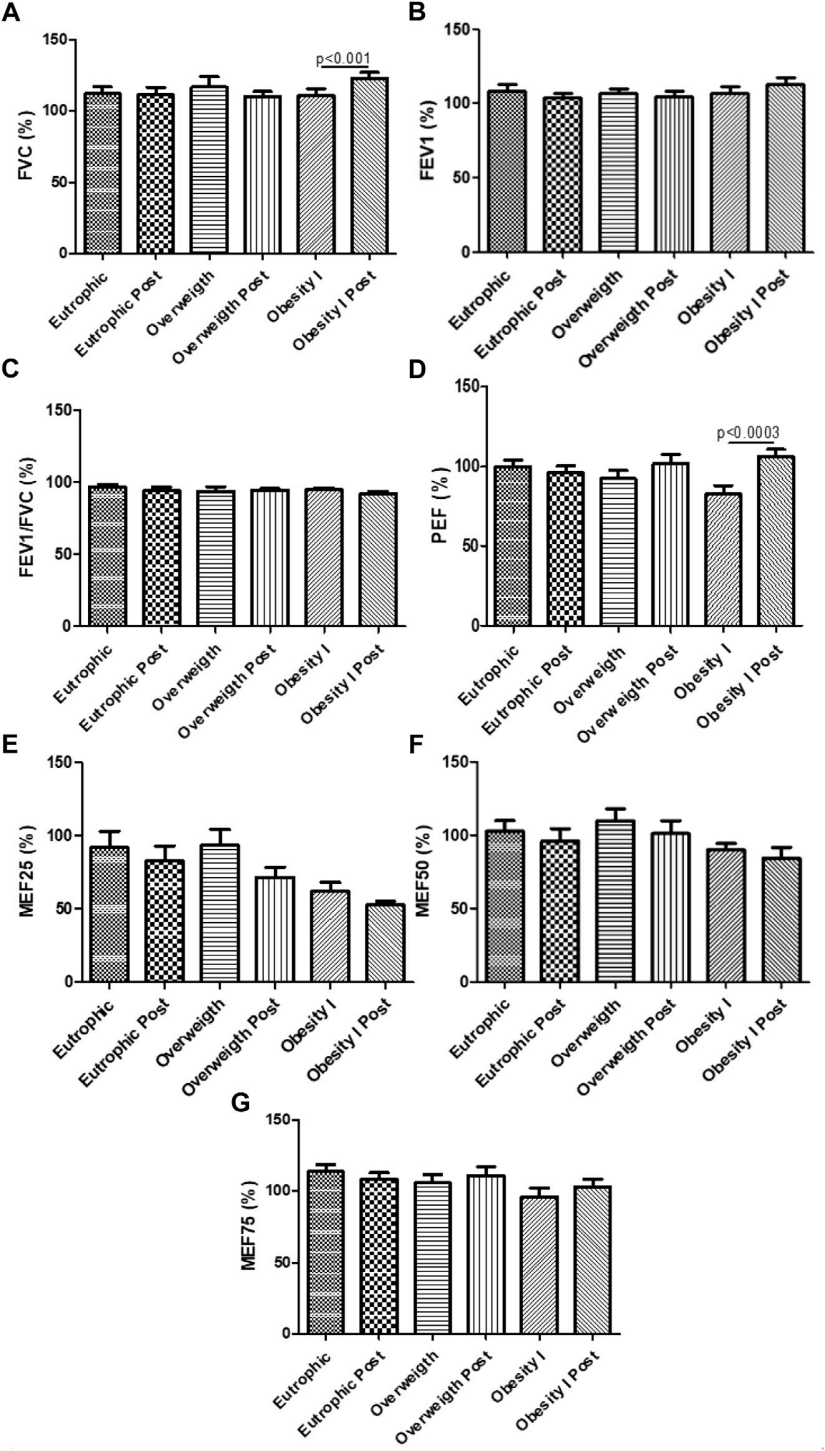
Effects of combined training on the lung function test. Figure 3A shows the FVC%; Figure 3B the VEF1%; Figure 3C the VEF1/FVC; Figure 3D PEF%; Figure 3E MEF25%; Figure 3F MEF50%; Figure 3G MEF75%. Paired *t*-test was performed to compare the pre- and post-effects of rehabilitation (i.e., eutrophic non-obese pre x eutrophic non-obese post; overweight pre x overweight post; obesity I pre x obesity I post). A *p* < 0.05 was considered statistically significant. These data presented parametric distribution and were additionally evaluated using one way ANOVA followed by the Newman–Keuls test for multiple comparisons among the groups.

Figure 3 shows the effects of combined training on lung function. Figure 3A shows that combined training improved the FVC% (*p* < 0.01) and the PEF% (*p* < 0.01) comparing the obesity I group pre- and post-training. All other parameters (VEF1, VEF1/FVC, MEF 25%, MEF 50%, and MEF 75%) of lung function obtained in the spirometry test did not present any differences.

### Effects of combined training on lung function and mechanics

**FIGURE 4:**
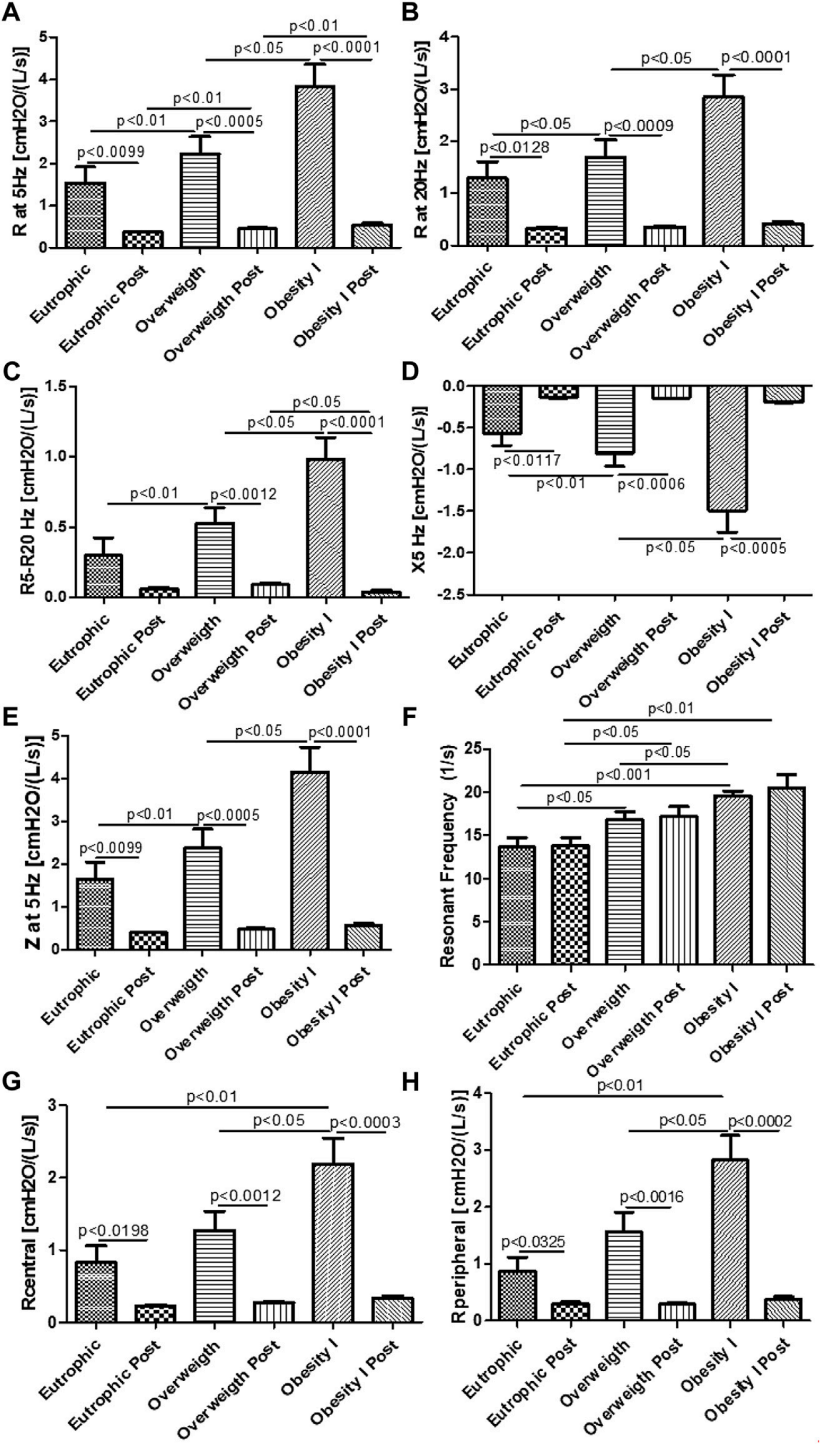
Effects of combined training on lung mechanics. Figure 4A shows the resistance of the whole respiratory system (R5 Hz); Figure 4B the resistance of the proximal airways (R20 Hz); Figure 4C the resistance of the distal airways (R5–20 Hz); Figure 4D the reactance of the respiratory systems (X5 Hz); Figure 4E the impedance of the respiratory systems (Z5 Hz); Figure 4F the resonant frequency (RFres); Figure 4G central resistance of the respiratory system (RCentral); Figure 4H the peripheral resistance of the respiratory system (RPeripheral). Paired *t*-test was performed to compare the pre- and post-effects of rehabilitation (i.e., eutrophic non-obese pre x eutrophic non-obese post; overweight pre x overweight post; obesity I pre x obesity I post). A *p* < 0.05 was considered statistically significant. These data presented parametric distribution and were additionally evaluated using one way ANOVA followed by the Newman–Keuls test for multiple comparisons among the groups.

Figure 4 shows the effects of combined training on lung mechanics. Figure 4A shows that combined aerobic training improved the resistance of the whole respiratory system (R5 Hz) for non-obese (*p* < 0.01), overweight (*p* < 0.01), and obesity I (*p* < 0.001) groups. Figure 4B shows that combined aerobic training improved the resistance of the proximal airways (R20 Hz) for non-obese (*p* < 0.05), overweight (*p* < 0.001), and obesity I (*p* < 0.001) groups. Figure 4C shows that combined aerobic training improved the resistance of the distal airways (R5 Hz–20 Hz) for non-obese (*p* < 0.01), overweight (*p* < 0.01), and obesity I (*p* < 0.001) groups. Figure 4D shows that combined aerobic training improved the reactance of the respiratory systems (X5 Hz) for non-obese (*p* < 0.05), overweight (*p* < 0.001), and obesity I (*p* < 0.01) groups. Figure 4E shows that combined aerobic training improved the impedance of the respiratory systems (Z5 Hz) for non-obese (*p* < 0.05), overweight (*p* < 0.01), and obesity I (*p* < 0.001) groups. Figure 4F shows that combined aerobic training did not change the resonant frequency (RFres). Figure 4G shows that combined aerobic training improved the central resistance of the respiratory systems (RCentral) for non-obese (*p* < 0.05), overweight (*p* < 0.01), and obesity I (*p* < 0.001) groups. Similarly, the peripheral resistance (RPeripheral) of the respiratory system was also improved by combined aerobic training for non-obese (*p* < 0.05), overweight (*p* < 0.01), and obesity I (*p* < 0.001) groups.

### Effects of combined training on pulmonary fibrotic biomarkers

**FIGURE 5:**
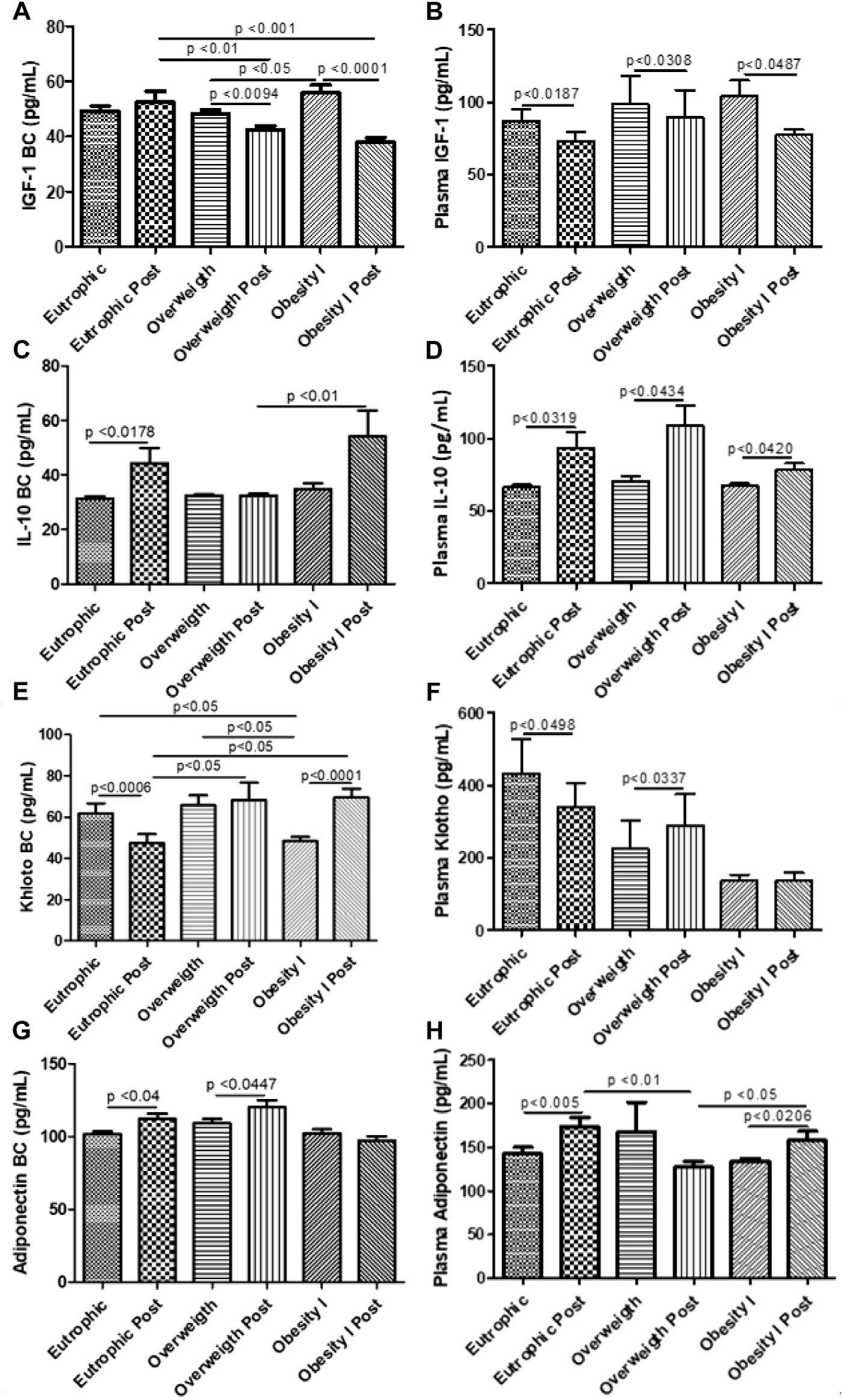
Effects of combined training on pulmonary and systemic fibrotic biomarkers. Figure 5A shows the pulmonary levels of IGF-1; Figure 5B the plasma levels of IGF-1; Figure 5C the pulmonary levels of the anti-inflammatory cytokine IL-10; Figure 5D the plasma levels of the anti-inflammatory cytokine IL-10; Figure 5E the pulmonary levels of anti-inflammatory and anti-fibrotic protein Klotho; Figure 5F the plasma levels of anti-inflammatory and anti-fibrotic protein Klotho; Figure 5G the pulmonary levels of the anti-inflammatory adipokine adiponectin; and Figure 5H the plasma levels of the anti-inflammatory adipokine adiponectin. Paired *t*-test was performed to compare the pre- and post-effects of rehabilitation (i.e., eutrophic non-obese pre x eutrophic non-obese post; overweight pre x overweight post; obesity I pre x obesity I post). A *p* < 0.05 was considered statistically significant. These data presented parametric distribution and were additionally evaluated using one way ANOVA followed by the Newman–Keuls test for multiple comparisons among the groups.

Figure 5 shows the effects of combined training on pulmonary fibrotic biomarkers. Figure 5A shows that combined training reduced the pulmonary levels of IGF-1 in overweight (*p* < 0.05) and obesity I (*p* < 0.001) groups but not in the non-obese group. Figure 5B shows that combined training reduced the plasma levels of IGF-1 in non-obese (*p* < 0.05), overweight (*p* < 0.05), and obesity I (*p* < 0.05) groups. Figure 1C shows that combined training increased the pulmonary levels of the anti-inflammatory cytokine IL-10 in non-obese (*p* < 0.05) and obesity I (*p* < 0.01) groups but not in the overweight group. Figure 5D shows that combined training increased the plasma levels of the anti-inflammatory cytokine IL-10 in non-obese (*p* < 0.05), overweight (*p* < 0.05), and in obesity I (*p* < 0.05) groups. Figure 5E shows that combined training decreased the pulmonary levels of anti-inflammatory and anti-fibrotic protein Klotho in the non-obese (*p* < 0.01) group, while increased its levels in the obesity I (*p* < 0.001) group without affecting the overweight group.

### Effects of combined training on systemic fibrotic biomarkers

Figure 5 shows the effects of combined training on systemic fibrotic biomarkers. Figure 5F shows that combined training increased the plasma levels of anti-inflammatory and anti-fibrotic protein Klotho in the overweight (*p* < 0.05) group, without affecting the non-obese and obesity I groups. Figure 5G shows that combined training increased the pulmonary levels of the anti-inflammatory adipokine adiponectin in non-obese (*p* < 0.05) and in overweight (*p* < 0.05) groups but not in the obesity I group. Figure 5H shows that combined training increased the plasma levels of the anti-inflammatory adipokine adiponectin in non-obese (*p* < 0.01) group and obesity I (*p* < 0.05) group but not in the overweight group.

### Effects of combined training on the systemic cellular immune response

**FIGURE 6:**
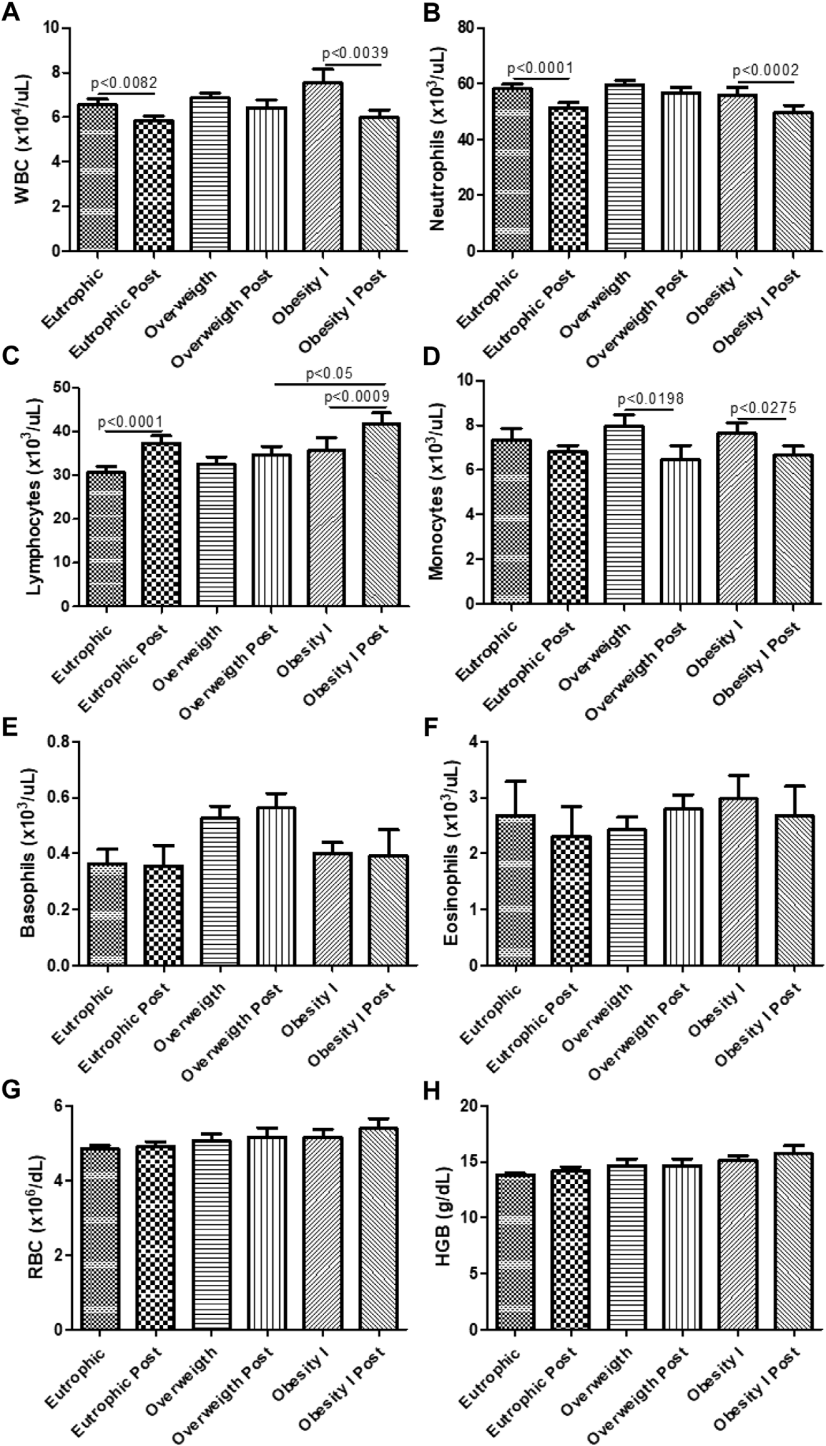
Effects of combined training on the systemic cellular immune response. Figure 6A shows the number of total leukocytes (white blood cells—WBC). Figure 6B shows the number of neutrophils. Figure 6C shows the number of lymphocytes. Figure 6D shows the number of monocytes. Figure 6E shows the number of basophils. Figure 6F shows the number of eosinophils. Figure 6G shows the number of red blood cells (RBC). Figure 6H shows the number of glycated hemoglobin (HBG). Paired *t*-test was performed to compare the pre- and post-effects of rehabilitation (i.e., eutrophic non-obese pre x eutrophic non-obese post; overweight pre x overweight post; obesity I pre x obesity I post). A *p* < 0.05 was considered statistically significant. These data presented parametric distribution and were additionally evaluated using one way ANOVA followed by the Newman–Keuls test for multiple comparisons among the groups.

Figure 6 shows the effects of combined training on the cellular immune response. Figure 6A shows that combined training reduces the number of leukocytes in non-obese (*p* < 0.01) and obesity I (*p* < 0.01) groups but not in the overweight group. The number of neutrophils was also reduced by combined training in non-obese (*p* < 0. 001) and obesity I (*p* < 0.001) groups but not in the overweight group as shown in Figure 6B. On the other hand, Figure 6C shows that combined training increased the number of lymphocytes in non-obese (*p* < 0. 001) and obesity I (*p* < 0.001) groups but not in the overweight group. The number of monocytes was also reduced by combined training in overweight (*p* < 0.05) and obesity I (*p* < 0.05) groups but not in the non-obese group as shown in Figure 6D. No differences were observed in the number of basophils (Figure 6E) and eosinophils (Figure 6F). In addition, the results demonstrated that no changes were observed for red blood cells (Figure 6G) and hemoglobin (Figure 6H).

## Discussion

The present study shows for the first time that 12 weeks of combined training protocol, using aerobic treadmill training plus resistance training reduces visceral fat and lung inflammation, improves respiratory muscle strength and functional capacity, and improves lung function and mechanics to reduce pulmonary and systemic inflammation in overweight and grade I obese women.

### Effects of fat distribution on lung function and mechanics

Two phenotypes of obesity have been described: 1) central obesity, characterized by increased visceral fat accumulation and 2) peripheral obesity, characterized by subcutaneous fat accumulation (Suárez-Cuenca et al., 2021). This difference in fat distribution is linked with the pathophysiological effects of obesity triggering the development of different comorbidities (Kwon et al., 2017). In this way, it has been demonstrated that increased visceral fat is more likely to be linked to asthma phenotype development and impaired lung function, also negatively impacting lung mechanics (Collins et al., 1995; Kwon et al., 2017; Dixon and Peters, 2018). In the present study, it was observed that not only obesity grade I but also being overweight negatively impacted lung mechanics, impairing airway, and tissue resistance and elastance; all the studied groups presented increased levels of visceral fat. Of note, the present study shows for the first time that combined training improved all parameters of lung mechanics, strongly suggesting a positive effect of combined training on lung structure and remodeling, which can be functionally reflected by alterations in lung mechanics. Such observations are important since they reveal that the pulmonary alterations induced by overweight and obesity can be attenuated by combined training. Furthermore, previous studies have demonstrated that the parameters measured in lung mechanics by impulse oscillometry are more sensible than lung function to detect the pulmonary alterations induced by obesity (Zerah et al., 1993; Oppenheimer et al., 2012).

### Immune mechanisms of exercise in the improvement of lung mechanics

In addition, the importance of the present study is guaranteed once it reveals that not only can lung mechanics be improved by combined training in overweight and obese women but also reveals the possible immunological mechanisms involved. In fact, IGF-1 is considered a key growth factor overproduced in asthma and obesity (Han et al., 2020). In asthma, IGF-1 modulates airway inflammation and hyper-responsiveness and airway smooth muscle hyperplasia, resulting in impaired lung mechanics (Lee et al., 2014). In addition, pre-clinical studies using mouse models of asthma showed that IGF-1 is upregulated in lung tissue, and the administration of IGF-1-neutralizing antibodies reduced airway resistance and inflammation (Yamashita et al., 2005). So, the present study has demonstrated that combined training not only reduced the increased plasma levels of IGF-1 in non-obese, overweight, and obesity I women but also reduced pulmonary IGF-1, as demonstrated in breath condensate. However, we are aware that the reduction of IGF-1 alone would not justify these observed effects. So, the present study demonstrated that combined training was able to increase the pulmonary and systemic levels of the anti-fibrotic and anti-aging protein Klotho (Mencke et al., 2017). In fact, although the present study used a chronic exercise regimen (12 weeks), other studies also have demonstrated that not only chronic (Matsubara et al., 2014) but also acute (Santos-Dias et al., 2017) exercise may upregulate the synthesis and release of Klotho, as observed in the present study. Of note, the study by Matsubara et al., 2014, demonstrated that chronic exercise increases plasma levels of Klotho, resulting in better functional arterial stiffness in postmenopausal women, an event with similar issues concerning the pathobiology of lung fibrosis (i.e., inflammation and smooth muscle proliferation and increased synthesis and accumulation of extracellular matrix proteins) (Mencke et al., 2017). So it is plausible to postulate that combined exercise training improved lung function and mechanics not only by reducing IGF-1 but also by increasing Klotho release.

### Exercise positively affects inflammation in overweight and obese women

Furthermore, obesity is characterized by sub-clinical inflammation, which encompasses increased levels of pro-inflammatory cytokines and reduced levels of anti-inflammatory cytokines (Tinius et al., 2020). In fact, some studies have demonstrated that different modalities of training, including acute and chronic response, can increase the systemic levels of anti-inflammatory cytokines, notably, the anti-inflammatory adipokine adiponectin and interleukin 10 (IL-10) (Soltani et al., 2020; Seo et al., 2021). In this way, the present study demonstrated that combined exercise training increased the plasma levels of adiponectin in non-obese and obesity grade I women and the plasma levels of IL-10 in non-obese, overweight, and obesity grade I women. Furthermore, the present study demonstrated for the first time that combined exercise training increased the pulmonary levels of adiponectin in non-obese and overweight women and the levels of IL-10 in non-obese and obesity grade I women, revealing that such anti-inflammatory effects of exercise training are not limited to the systemic circulation, but also reaches the lungs, counter-parting the effects of overweight and obesity.

### Exercise reduces pulmonary nitric oxide production in overweight and obese women

Beyond the imbalance of pro- and anti-inflammatory cytokines, some studies demonstrated that overweight and obesity are followed by increased levels of fractional exhaled nitric oxide (FeNO) (Chow et al., 2009; Van de Kant et al., 2016; Al Khathlan and Salem, 2020), which is associated with the inflammatory response of the airways in obese women (Carpagnano et al., 2008). In the present study, it was also found that both overweight and obesity grade I women presented increased levels of FeNO. In addition, increased levels of FeNO are associated also with airway hyper-responsiveness and impaired lung mechanics, notably, increased airway resistance (Almeida et al., 2020; Lajunen et al., 2020). Similarly, the present study demonstrated that both overweight and obese grade I women displayed impaired lung mechanics, such as increased resistance of the whole respiratory system (R5 Hz), increased resistance of the proximal airways (R20 Hz), increased resistance of the small/distal airways (R5–R20 Hz), increased impedance of the lungs (Z5 Hz), increased reactance of the respiratory system (X5 Hz), resonant frequency (RFres), and central (RCentral) and peripheral (RPeripheral) resistance of the respiratory system. So, this study shows for the first time that combined training reduces FeNO in overweight and obese grade I women, resulting in improved lung mechanics.

### Exercise improves respiratory muscle strength and functional capacity in overweight and obese women

In addition, the literature clearly demonstrates that impaired lung mechanics in obese individuals is partially mediated by excessive fat accumulation in the thorax, limiting the function of respiratory muscles, which leads to fatigue and low strength (Mafort et al., 2016). However, many studies demonstrate that obese individuals may not present decreased maximal inspiratory pressure (MIP) and maximal expiratory pressure (MEP) (Mafort et al., 2016; Sant'Anna et al., 2019). In this way, the present study demonstrated that overweight and obese grade I women did not present reduced MIP and MEP, but, anyway, that combined training significantly improved the MIP and MEP in overweight and obese grade I women, which may be another factor involved in the improvement of lung mechanics observed in this study. Finally, obesity is related to decreased levels of activity and exercise capacity, which can be investigated by using the incremental shuttle walking test (Peixoto-Souza et al., 2015). In this way, the present study demonstrated that although no differences were found before the beginning of combined training in non-obese, overweight, and obese grade I women, the combined training improved the exercise capacity and tolerance, as demonstrated by increased distance reached in the incremental shuttle walking test, demonstrating that the systemic and the pulmonary anti-inflammatory effects and the improvement in lung mechanics and respiratory muscles induced by combined training by the end also resulted in improved exercise capacity.

## Conclusion

Therefore, we conclude that combined training is effective in reducing visceral fat, systemic and pulmonary inflammation and fibrotic biomarkers, and to improve physical capacity in non-obese, overweight, and obese grade I middle-aged women.

## Data Availability

The raw data supporting the conclusion of this article will be made available by the authors, without undue reservation.
